# Body composition and grip strength are improved in transgenic sickle mice fed a high-protein diet

**DOI:** 10.1017/jns.2014.63

**Published:** 2015-02-27

**Authors:** Patrice L. Capers, Hyacinth I. Hyacinth, Shayla Cue, Prasanthi Chappa, Tatyana Vikulina, Susanne Roser-Page, M. Neale Weitzmann, David R. Archer, Gale W. Newman, Alexander Quarshie, Jonathan K. Stiles, Jacqueline M. Hibbert

**Affiliations:** 1 Departments of Microbiology, Biochemistry and Immunology/Medicine, Morehouse School of Medicine, 720 Westview Drive SW, Atlanta, GA 30310, USA; 2 University of Alabama at Birmingham, 1720 2nd Avenue South, Birmingham, AL 35294, USA; 3 Medical University of South Carolina, 169 Ashley Avenue, SC 29403, USA; 4 Aflac Cancer and Blood Disorder Center, Children's Healthcare of Atlanta, Emory University, 2015 Uppergate Drive, Atlanta, GA 30322, USA; 5 Division of Endocrinology and Metabolism and Lipids, Emory University School of Medicine, 101 Woodruff Circle, 1305 WMRB, Atlanta, GA 30322, USA; 6 Atlanta VA Medical Center, 1670 Clairmont Road, Decatur, GA 30033, USA

**Keywords:** High-protein diet, Sickle cell disease, Grip strength, Body composition, BMC, bone mineral content, BMD, bone mineral density, C, C57BL/6 (control) mice, C20, control mice fed diet supplying 20 % energy from protein, C35, control mice fed diet supplying 35 % energy from protein, DXA, dual-energy X-ray absorptiometry, l-Arg, l-arginine, LBM, lean body mass, S, Berkeley transgenic sickle mice, S20, Berkeley sickle mice fed diet supplying 20 % energy from protein, S35, Berkeley sickle mice fed diet supplying 35 % energy from protein, SCA, sickle cell anaemia, TS, Townes sickle mice, TS0.8, Townes sickle mice fed 0·8 % l-Arg diet, TS1.6, Townes sickle mice fed 1·6 % l-Arg diet, TS3.2, Townes sickle mice fed 3·2 % l-Arg diet, TS6.4, Townes sickle mice fed 6·4 % l-Arg diet

## Abstract

Key pathophysiology of sickle cell anaemia includes compensatory erythropoiesis, vascular injury and chronic inflammation, which divert amino acids from tissue deposition for growth/weight gain and muscle formation. We hypothesised that sickle mice maintained on an isoenergetic diet with a high percentage of energy derived from protein (35 %), as opposed to a standard diet with 20 % of energy derived from protein, would improve body composition, bone mass and grip strength. Male Berkeley transgenic sickle mice (S; *n* 8–12) were fed either 20 % (S20) or 35 % (S35) diets for 3 months. Grip strength (BIOSEB meter) and body composition (dual-energy X-ray absorptiometry scan) were measured. After 3 months, control mice had the highest bone mineral density (BMD) and bone mineral content (BMC) (*P* < 0·005). S35 mice had the largest increase in grip strength. A two-way ANOVA of change in grip strength (*P* = 0·043) attributed this difference to genotype (*P* = 0·025) and a trend in type of diet (*P* = 0·067). l-Arginine (l-Arg) supplementation of the 20 % diet was explored, as a possible mechanism for improvement obtained with the 35 % diet. Townes transgenic sickle mice (TS; *n* 6–9) received 0·8, 1·6, 3·2 or 6·4 % l-Arg based on the same protocol and outcome measures used for the S mice. TS mice fed 1·6 % l-Arg for 3 months (TS1.6) had the highest weight gain, BMD, BMC and lean body mass compared with other groups. TS3.2 mice showed significantly more improvement in grip strength than TS0·8 and TS1.6 mice (*P* < 0·05). In conclusion, the high-protein diet improved body composition and grip strength. Outcomes observed with TS1.6 and TS3.2 mice, respectively, confirm the hypothesis and reveal l-Arg as part of the mechanism.

Sickle cell anaemia (SCA) is a genetic disorder of Hb, affecting the structure and function of erythrocytes. In response to certain physiological conditions, such as hypoxia, erythrocytes assume a sickled shape and become less adaptable^(^^1^^,^^2^^)^. These abnormal erythrocytes adhere to vessels and restrict blood flow, causing endothelial injuries, vaso-occlusive crises associated with pain, and ultimate end organ damage^(^^3^^)^. Together, subclinical endothelial injury, erythropoiesis, transient vaso-occlusive events and increased intra-vascular haem from haemolysis promote steady-state inflammation in SCA patients^(^^4^^,^^5^^)^.

Haemolysis catalyses the generation of reactive oxygen species, which decrease NO availability. In the body, l-arginine (l-Arg) is an amino acid required for protein synthesis, urea and NO production^(^^6^^)^. The functions of l-Arg are many, including growth and muscle development^(^^6^^–^^8^^)^, making it a semi-essential amino acid based on the stage of development. Both mice^(^^9^^–^^11^^)^ and human subjects^(^^12^^,^^13^^)^ with sickle cell disease typically have low Arg levels associated with vasoconstriction and several attendant complications, including acute chest syndrome. In SCA, Arg metabolism is shifted towards increased urea production^(^^14^^)^, limiting NO production. The increased presence of haem also scavenges NO leading to vasoconstriction promoting hypoxia and organ damage^(^^15^^)^ and causing an increase in proinflammatory markers.

The inflammatory response is associated with hypermetabolism^(^^4^^)^ and muscle proteolysis^(^^16^^–^^18^^)^. During muscle proteolysis^(^^16^^,^^17^^)^ pro-inflammatory IL-6 initiates the synthesis of acute-phase proteins, which require increased amino acid uptake^(^^19^^)^ to further propagate the chronic inflammatory response. Increased energy demands of haemolysis, inflammation and other steady-state complications affect the growth and development of SCA patients^(^^20^^,^^21^^)^. Children with SCA have significantly lower body weight, height, bone mineral density (BMD) and bone mineral content (BMC) compared with healthy controls^(^^22^^–^^24^^)^. These processes increase the nutritional requirements for SCA patients, making an otherwise normal dietary intake insufficient^(^^25^^)^ to maintain growth and development, as often observed among SCA patients^(^^20^^,^^26^^)^.

This idea is supported by findings from our previous work examining a series of diets with 15–35 % energy from protein, where sickle mice maintained on a 35 % energy from protein diet increased weight gain and decreased baseline inflammatory indicators, C-reactive protein and IL-6 and liver arginase activity^(^^11^^)^. An important next step was to examine the impact of the diet on body composition, since the original premise that the increased proportion of energy derived from protein would promote weight gain had been confirmed^(^^11^^)^. Our hypothesis was that sickle mice maintained on a test diet with a high proportion of energy supplied as protein (35 %) *v.* a standard diet with 20 % energy supplied as protein would improve body composition and improve bone structure and grip strength, while sustaining erythropoietic activity. Berkeley transgenic sickle mice (S mice) developed by Pászty *et al.*^(^^27^^)^ were used for the present study because only human α- and sickle β-globins are transgenically expressed in these mice, providing a suitable *in vivo* model to examine salient characteristics of clinical SCA. It was also considered that l-Arg could have a role in any improvement in body composition observed, due to increased l-Arg availability from increased dietary protein, for muscle protein synthesis, tissue replacement and repair.

The focus of the present research was therefore to investigate the effect of the high-protein diet on body composition, including bone mass and grip strength. The pattern of these outcomes would then be used as a guide for determining expected outcomes when investigating a possible role for increased l-Arg availability from the high-protein diet. The l-Arg effect was determined by supplementing the standard 20 % energy from protein diet with increasing doses of l-Arg, to determine if there was also a dose–response effect on the outcome measures. We hypothesised that increasing the amount of l-Arg in the diet, would improve body composition and grip strength beyond that achieved by sickle mice maintained only on the standard diet. Confirmation of this hypothesis would suggest a role for l-Arg as a component of the high-protein diet, in facilitating physiological changes in body composition. Townes^(^^28^^,^^29^^)^ sickle (TS) mice were used to examine l-Arg supplementation, and, like S mice^(^^27^^)^, express human sickle Hb exclusively, erythrocyte sickling, severe anaemia and progressive organ pathology as in humans with SCA^(^^27^^–^^30^^)^.

## Experimental methods

### Mice

Male S mice (*n* 8–12) were used in a prospective controlled terminal feeding trial. The S mouse model is derived from a mixed genetic background (FVB/N, 129, DBA/2, C57BL/6, Black Swiss)^(^^27^^)^. C57BL/6 mice (C; *n* 8–12) were therefore used as controls. Whereas laboratory mice generally grow optimally on a 20 % energy from protein diet, our preliminary studies confirmed that sickle mice grew best on a 35 % energy from protein diet^(^^11^^)^. Therefore for the first aim of the present study we compared the effect of a 35 % energy from protein diet with a 20 % energy from protein diet on body composition of both C and S mice. TS mice (*n* 6–9) were utilised for the second aim^(^^28^^)^ to investigate the effect of l-Arg supplementation because our collaborators were switching from the Berkeley colony to the Townes model. Both models^(^^27^^,^^28^^)^ resulted from shared breeding, by two research groups, of a knockout murine α-model with a β-globin model. The research groups independently bred the resulting model with both murine knockouts, to developed mice carrying human transgenes^(^^31^^)^. Both models express similar human sickle Hb pathology and were appropriate for the outcomes investigated in the present study.

Weanling mice (aged about 4 weeks old) were typically housed four per cage for 1 week of acclimatisation followed by 3 months of feeding. Specially designed cages, separating wasted food crumbs from urine, faeces and bedding were used to allow calculation of the actual amount of feed consumed by the mice per cage. All guidelines for the care and use of animals were followed and The Institutional Animal Care and Use Committees of Emory University and Morehouse School of Medicine approved all experimental procedures.

### Study design

The study was designed as a prospective controlled terminal feeding trial. The requirement of eight mice per group was based on an 80 % power calculation with an α of 0·05. For all feeding experiments mice were randomly assigned to any of the selected diets. After weaning, the mice were allowed to acclimatise to their diets: standard 20 % or test 35 % diet and 0·8 % l-Arg, 1·6 % l-Arg, 3·2 % l-Arg, or 6·4 % l-Arg for 1 week.

Diets were supplied by Purina Mills TestDiet Division. For the diet supplying 20 % energy as protein, diet TD 1813657 was used, which contained (g/kg diet): vitamin-free casein, 223·0; dextrin, 353·0; l-Arg, 8·0; energy (kJ/kg diet), 15271·6. For the diet supplying 35 % energy as protein, diet TD 1813675 was used, which contained (g/kg diet): vitamin-free casein, 392·0; dextrin, 184·0; l-Arg, 13·7; energy (kJ/kg diet), 14853·2. Identical components were: sucrose, 157·0; glucose, 107·0; maize oil, 40·0; powdered cellulose, 50·0; American Institute of Nutrition (AIN) 93M mineral mix, 10·0; l-cystine, 3·0; choline bitartrate, 2·0.

For the 0·8 % l-Arg diet, diet TD 1813657 was used (g/kg diet): dextrin, 353·0; l-Arg, 8·0; energy (kJ/kg diet), 15271·6. For the 1·6 % l-Arg diet, diet TD 1813672 was used (g/kg diet): dextrin, 343·0; l-Arg, 16·0; energy (kJ/kg diet), 15230·0. For the 3·2 % l-Arg diet, diet TD 1813673 was used (g/kg diet): dextrin, 324·0; l-Arg, 32·0; energy (kJ/kg diet), 15188·0. For the 6·4 % l-Arg diet, diet TD 1813674 was used (g/kg diet): dextrin, 285·0; l-Arg, 64·0; energy (kJ/kg diet), 15062·0. Identical components were: vitamin-free casein, 223·0 and those previously stated for the 20 % and 35 % protein diets.

All mice were then fed *ad libitum* for 3 months and monitored daily to ensure general health. Three mice died as a result of sickle cell complications in the 3·2 % l-Arg treatment group. The information collected before death was used in the analysis of food intake. Food consumption corrected for spillage was recorded and weekly body weights were measured. Body composition was determined by dual-energy X-ray absorptiometry (DXA) scan, and grip strength was measured 0 to 3 d before the end of the feeding period, using a transducer (both are designed for mice and detailed below). Blood was also collected via tail clip to measure complete blood count using a veterinary haematology analyser (Hematrue™; HESKA Lab Systems) and reticulocyte count via flow cytometry (BD LSR II; BD Biosciences). After the DXA scan, the mice were killed by isoflurane anaesthesia followed by cervical dislocation. Blood was then obtained by cardiac puncture and stored for future use.

### Grip strength

At the end of the feeding period, a validated grip strength test meter (BIOSEB; EB Instruments) was used to measure the grip strength of all limbs^(^^32^^)^. Grip strength was also recorded before the start of the feeding period after 1 week of acclimatisation to the diet. During the grip strength test, the mice were handled by their tails and placed over the grid until all paws grasped the grid. The tail was then pulled horizontally until the mouse released hold entirely. Three separate readings were recorded and averaged in Newtons, then converted to grams for analysis. Change in grip strength was calculated by the difference between the initial value after acclimatisation and the final value at 3 months.

### Body composition

Body composition was determined *in vivo* using a validated DXA instrument for mice (Lunar PIXImus2 Densitometer; GE Medical Systems)^(^^33^^)^. DXA scans were performed only once to reduce risk of death for mice recovering from anaesthesia. Mice were anaesthetised using a ketamine (100 mg/kg)–xylazine (10 mg/kg) mixture and positioned right side up on the plate. Whole-body DXA was performed, which provided data for BMD (amount of mineral in bone within a certain volume), BMC (the weight of minerals within bone), percentage fat (the percentage of fat in the whole body) and lean body mass (LBM, the amount of lean mass in the whole body). The long-term inter-assay CV for this technique is 0·65 %.

### Weight gain

The mice were weighed before feeding commenced and the total weight gain was measured by subtracting the final from the initial weight. The total weight of food supplied to the mice over the study period was corrected for spillage and the quantity of food consumed per cage was determined. Since the mice were not individually caged we added their weights per cage to determine changes in weight gain in response to quantity of food consumed. Weekly weight per cage was divided by weekly food consumption and the results were plotted to illustrate differences in weight gain by type of diet.

### Statistical analysis

Statistical testing of normality for continuous variables revealed abnormal distributions. Differences between groups were therefore analysed using the non-parametric Mann–Whitney test and the values are presented as mean values and standard deviations. To determine the effect of the high-protein diet, a two-way ANOVA model of either total weight gain or change in grip strength as outcome variables on mouse genotype, protein level, and mouse genotype × protein level (the interaction term) was performed. Kruskal–Wallis with a *post hoc* (Mann–Whitney) test was used to compare the differences between mice on the l-Arg diet. We also compared S20 and TS mice fed a 0·8 % l-Arg diet (TS0.8) to determine the effect of type of transgenic mouse model on total weight gain, change in grip strength, and body composition. The *P* values for the models were resolved from the *F* tests and *P* values < 0·05 were considered as significant for all statistical tests. Analyses were conducted using IBM SPSS Statistics v22.0 (IBM Corp.) and GraphPad Prism v5.0 (GraphPad Software, Inc.) statistical software packages.

## Results

### Effect of high-protein diet on body composition

#### Characteristics of mice

**Fig. 1.**
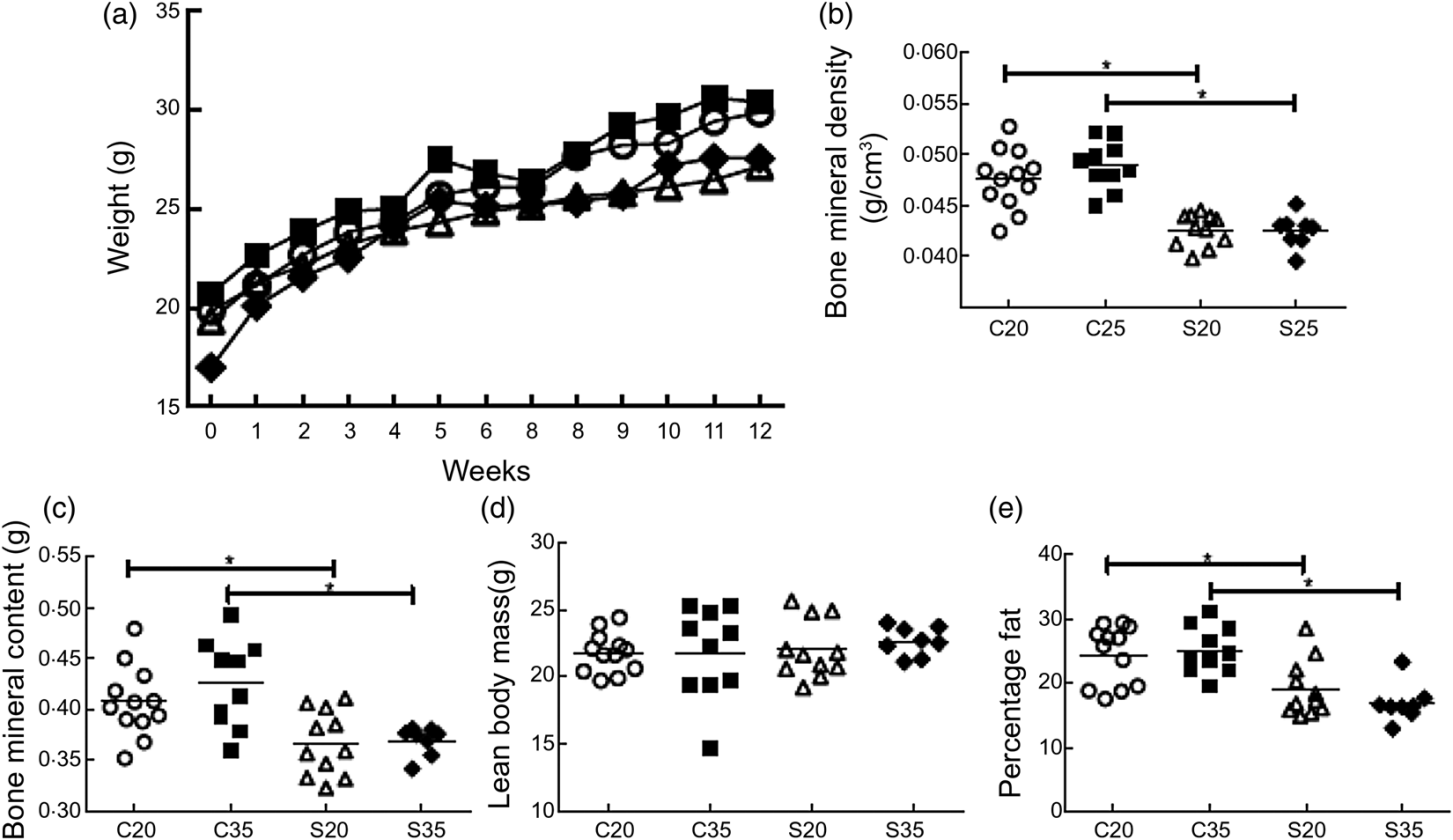
Effect of diet on body composition of sickle and control mice fed either 20 or 35 % of energy from protein for 3 months. Weight values represent the mean weekly weight per group (a). Bone mineral density (b) and bone mineral content (c) of control mice were significantly higher than for sickle mice regardless of diet. Lean body mass (d) was not different across the groups. Sickle mice had a significantly lower percentage of fat than control mice (e). Body composition was plotted individually and the mean value for all mice represented by the horizontal line. C20, control mice fed a diet supplying 20 % energy from protein (○); C35, control mice fed a diet supplying 35 % energy from protein (■); S20, Berkeley sickle mice fed a diet supplying 20 % energy from protein (△); S35, Berkeley sickle mice fed a diet supplying 35 % energy from protein (◆). * *P* < 0·05.

After 3 months on the diets, mean age range for the groups (C mice fed the diet supplying 20 % energy from protein (C20), C mice fed the diet supplying 35 % energy from protein (C35), S mice fed the diet supplying 20 % energy from protein (S20), S mice fed the diet supplying 35 % energy from protein (S35)) was 118–120 d. The typical characteristics of the S *v.* C mice, wherein S mice have lower Hb and higher reticulocyte and leucocyte counts, were seen and are mentioned elsewhere^(^^34^^)^. As expected, weight increased for all groups after 3 months of feeding (Fig. 1(a)). A two-way ANOVA model for total weight gain was not significant (*F* = 1·279; *P* = 0·296).

#### Body composition and grip strength

**Fig. 2.**
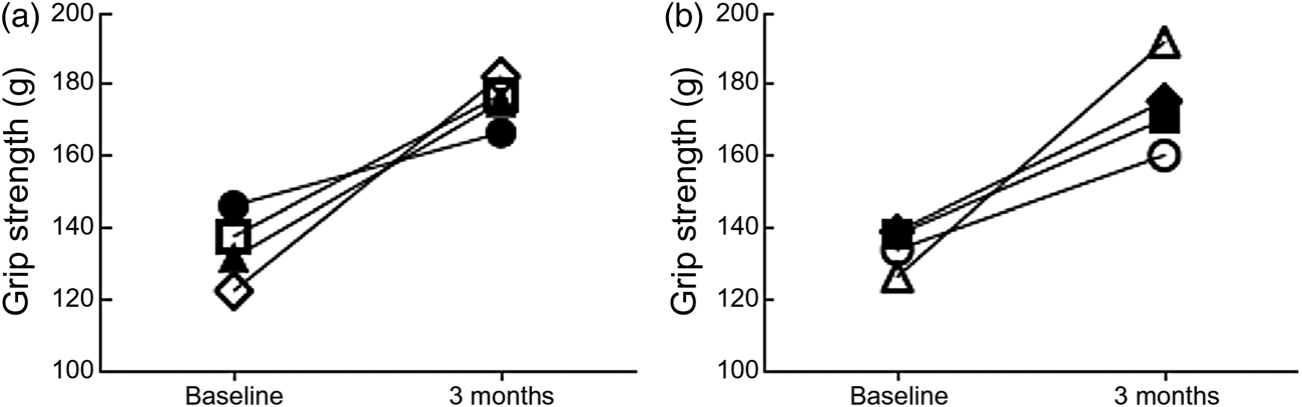
Grip strength after 3 months of feeding either 20 or 35 % of energy from protein or l-arginine (l-Arg) supplement. Grip strength improved after 3 months of feeding for all groups. The values are means illustrating baseline and final grip strength for each group. The S35 mice had the largest increase in grip strength over time in the standard *v.* high-protein diet (a). The TS3.2 mice had the largest increase in grip strength over time from l-Arg supplementation (b). ●, C20, control mice fed a diet supplying 20 % energy from protein; □, C35, control mice fed a diet supplying 35 % energy from protein; ▲, S20, Berkeley sickle mice fed a diet supplying 20 % energy from protein; ◊, S35, Berkeley sickle mice fed a diet supplying 35 % energy from protein; ○, TS0.8, Townes sickle mice fed 0·8 % l-Arg diet; ■, TS1.6, Townes sickle mice fed 1·6 % l-Arg diet; △, TS3.2, Townes sickle mice fed 3·2 % l-Arg diet; ◆, TS6.4, Townes sickle mice fed 6·4 % l-Arg diet.

BMD and BMC improved after 3 months of feeding. The BMD and BMC for C mice, regardless of diet, were significantly higher than for S mice at 3 months (*P* ≤ 0·011; Fig. 1(b) and (c)). S mice regardless of diet had significantly lower percentage fat than C mice (*P* < 0·001) at 3 months. A separate set of mice was fed the respective diet for 1 week and then body composition was measured. Comparing these values with the 3-month values, BMD and BMC were higher for C mice than S mice. Also, mice fed the 35 % diet had higher increases in BMD, BMC and LBM than those fed the 20% diet (Table 1). After the 3-month feeding period, grip strength increased the most among the S35 mice (by 59·9 g), followed by S20 (43·6 g), C35 (39·4 g) and C20 mice (20·4 g; Fig. 2(a)), even after controlling for food consumed. A two-way ANOVA model of the effect of genotype and protein level on change in grip strength (*P* = 0·043) demonstrated a significant main effect of genotype (*P* = 0·025) and a trend in type of diet (*P* = 0·067; Table 2).

**Table 1.** Body composition of mice fed either the standard or high-protein diet (Mean values and standard deviations)

	C20*		C20 3 months		C35*		C35 3 months		S20*		S20 3 months		S35*		S35 3 months	
	Mean	sd	Mean	sd	Mean	sd	Mean	sd	Mean	sd	Mean	sd	Mean	sd	Mean	sd
BMD (g/cm^3^)	0·033	0·001	0·048	0·003	0·032	0·001	0·049	0·002	0·036	0·004	0·043	0·002	0·032	0·004	0·042	0·002
BMC (g)	0·216	0·014	0·407	0·035	0·197	0·023	0·425	0·043	0·243	0·067	0·367	0·032	0·186	0·039	0·369	0·014
LBM (g)	14·23	0·93	21·78	1·49	12·35	0·60	21·78	3·41	14·10	2·04	22·01	2·15	12·00	1·84	22·64	1·07
Percentage fat	16·14	1·04	24·42	4·57	16·13	1·59	25·13	3·68	19·96	2·86	16·98	1·73	18·60	2·00	16·84	2·95

C20, control mice fed a diet supplying 20 % energy from protein; C35, control mice fed a diet supplying 35 % energy from protein; S20, Berkeley sickle mice fed a diet supplying 20 % energy from protein; S35, Berkeley sickle mice fed diet supplying 35 % energy from protein; BMD, bone mineral density; BMC, bone mineral content; LBM, lean body mass; DXA, dual-energy X-ray absorptiometry.

* Values represent a separate set of mice fed the respective diet for 7 d after which the DXA scan was performed. Values at 3 months represent mean DXA measurements recorded after 3 months of feeding. Comparison of the two values will provide a sense of expected increases in body composition with feeding over 3 months.

**Table 2.** Effect of mouse type (sickle or control), diet (protein level) and their interaction on change in grip strength*

	Partial SS	df	MS	*F*	*P*
Model	7882·812	3	2627·604	2·995	0·043
Genotype	4773·366	1	4773·366	5·440	0·025
Protein level	3134·118	1	3134·118	3·572	0·067
Genotype × protein level	18·206	1	18·206	0·021	0·886

SS, sum of squares; MS, mean square.

* Results of two-way ANOVA.

### Effect of arginine supplementation on weight gain and body composition

#### Characteristics of mice

**Fig. 3.**
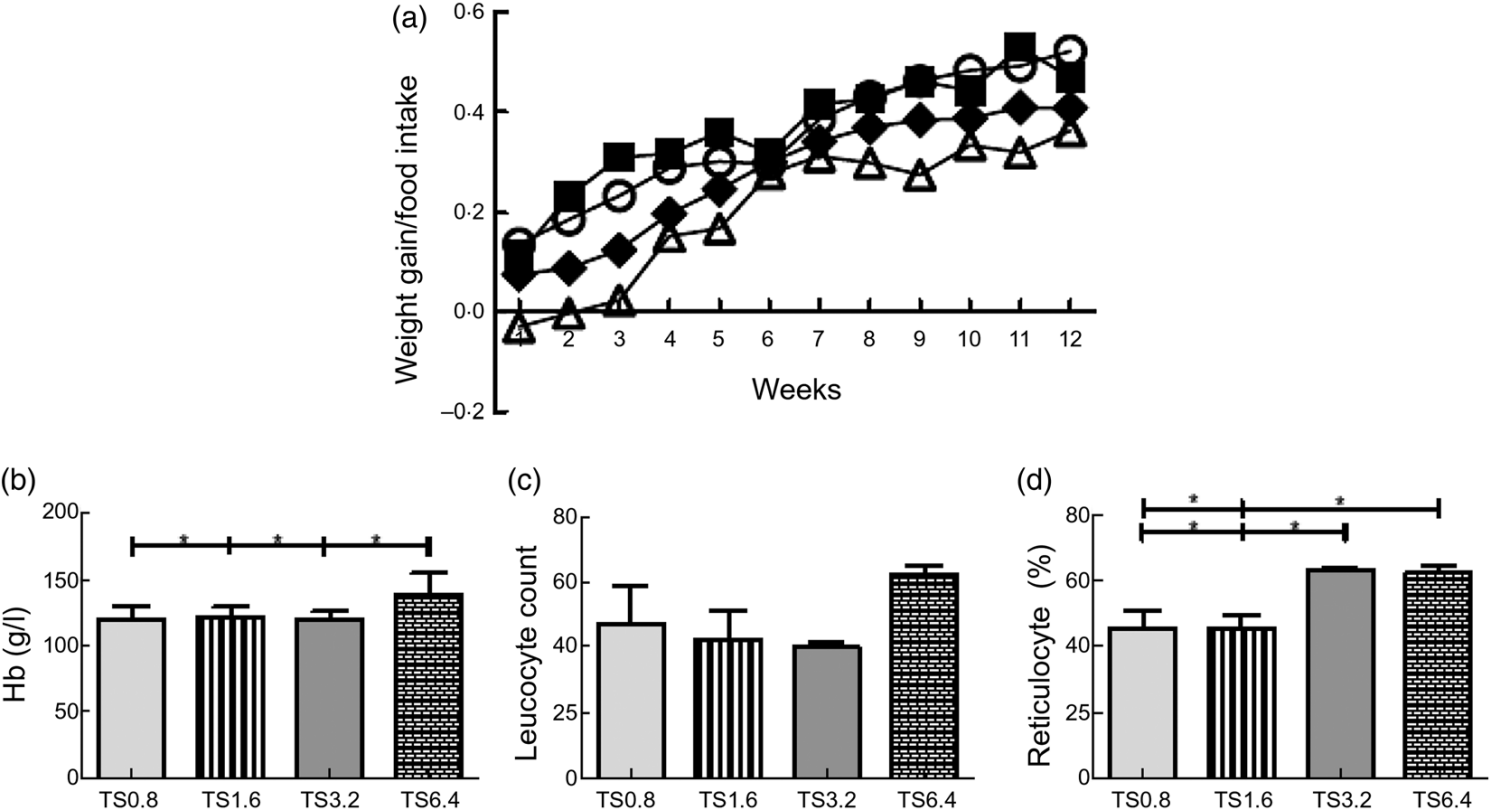
Effect of l-arginine (l-Arg) supplementation on weight and haematological parameters. Weight adjusted for food intake increased each week (a). For the majority of the 12 weeks the TS1.6 mice had the highest values followed by TS0.8 mice. The TS3.2 group (three of which died) had the lowest weight gain values. TS6.4 mice had significantly higher Hb levels than all other groups (b). No differences were observed for leucocyte count across groups (c). TS3.2 and TS6.4 mice had significantly higher reticulocyte percentages than TS0.8 and TS1.6 mice (d). ○, TS0.8, Townes sickle mice fed 0·8% l-Arg diet; ■, TS1.6, Townes sickle mice fed 1·6 % l-Arg diet; △, TS3.2, Townes sickle mice fed 3·2 % l-Arg diet; ◆, TS6.4, Townes sickle mice fed 6·4 % l-Arg diet. * *P* < 0·05.

Mean age after 3 months on the diets was 121–123 d. Kruskal–Wallis testing established significant differences in Hb (*P* = 0·038) and reticulocytes (*P* < 0·001). *Post hoc* analysis showed that TS mice fed a 6·4 % l-Arg diet (TS6·4) had significantly higher Hb than all groups (*P* ≤ 0·044; Fig. 3(b)). The reticulocyte percentages for the TS mice fed a 3·2 % l-Arg diet (TS3.2) and TS6.4 mice were also significantly higher than for both TS0.8 (*P* = 0·001, *P* < 0·001, respectively) and TS mice fed a 1·6 % l-Arg diet (TS1.6) (*P* = 0·001, *P* < 0·001; Fig. 3(d)).

#### Weight, rate of weight gain and grip strength

The TS1.6 mice had the lowest baseline weight. However, the final weight for this mouse group after 3 months of l-Arg supplementation was the highest among all mice receiving the four levels of dietary l-Arg (Fig. 3(a)). A similar pattern was observed with the S mice, in which the S35 mice receiving 1·6 g Arg/100 g of diet had the highest total weight gain after the 3-month feeding period. *Post hoc* analysis revealed that the total weight gain for TS1.6 mice was significantly higher than for TS6.4 mice (*P* = 0·047) and trended higher than for TS3.2 mice (*P* = 0·077), although the Kruskal–Wallis test was not significant (*P* = 0·094). Besides, the TS1.6 group typically had higher weekly weight gain values after adjusting for food intake (Fig. 3). Therefore, the average weekly weight gain/food intake over the 3-month period was higher for TS1.6 mice. Change in grip strength was significantly different between groups (*P* = 0·022) and *post hoc* analysis revealed significantly higher change for TS3.2 mice compared with TS0.8 (*P* = 0·008) and TS1.6 (*P* = 0·011) mice (Fig. 2(b)).

#### Body composition

**Fig. 4.**
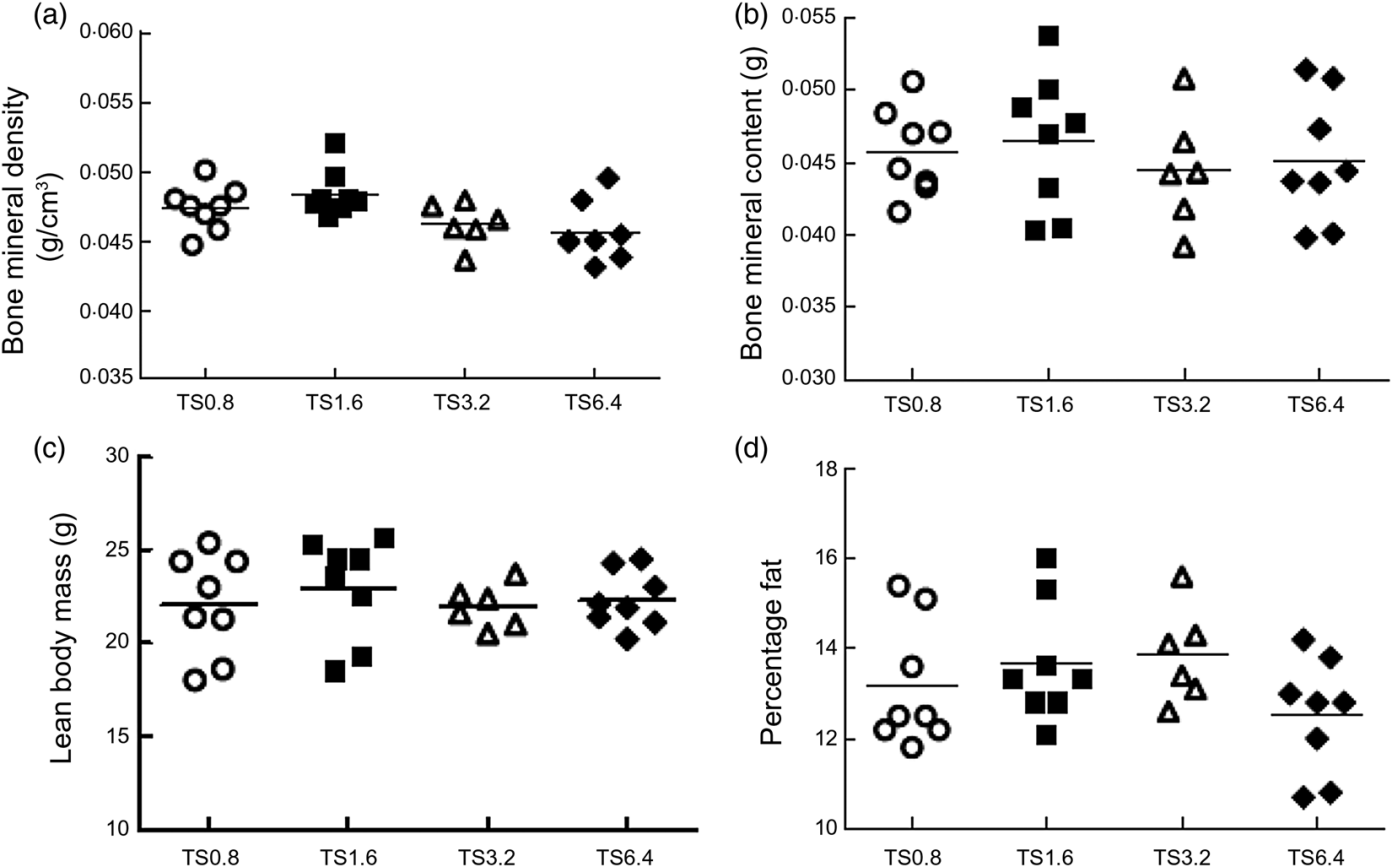
Effect of l-arginine (l-Arg) diets on body composition. Weight increased over 3 months of feeding. TS1.6 mice had highest mean weight at 3 months and showed the greatest improvement in weight compared with 0 weeks (baseline). Body composition improved with prolonged feeding. TS1.6 mice had the highest bone mineral density (a), bone mineral content (b) and lean body mass (c), while TS6.4 mice had the lowest percentage fat (d). For body composition individual values are plotted and the mean value is represented by the horizontal line. TS0·8, Townes sickle mice fed 0·8 % l-Arg diet; TS1.6, Townes sickle mice fed 1·6 % l-Arg diet; TS3.2, Townes sickle mice fed 3·2 % l-Arg diet; TS6.4, Townes sickle mice fed 6·4 % l-Arg diet.

BMD of TS1.6 mice was significantly higher than TS3.2 (*P* = 0·039) and tended to be higher than TS6.4 (*P* = 0·070) mice (Fig. 4) although the Kruskal–Wallis test was not significant (*P* = 0·128). As a reference for the 3-month l-Arg supplementation we fed age-matched TS mice (*n* 3) the 0·8 % l-Arg control diet for 1 week, and measured body composition. We chose not to perform this baseline measurement on additional diets because the main interest was in the outcome after 3 months of supplementing the control diet with l-Arg. The mean values from the DXA scan before supplementation were: BMD 0·034 (sd 0·002) g/cm^3^; BMC = 0·200 (sd 0·048) g; LBM 15·80 (sd 0·96) g; and percentage fat 14·17 (sd 2·51). Comparing these values with 3-month results, TS1.6 mice had higher body composition values in all components and TS3.2 mice had higher percentage fat. Comparison of the two mouse models (S *v.* TS) demonstrated that before supplementation TS0·8 mice had significantly higher BMD and BMC (*P* < 0·001) than S20 mice while S20 mice had significantly higher percentage fat (*P* = 0·001). However, the overall pattern of change during the experiments was similar for both models. Each intervention, i.e. increasing the proportion of energy derived from protein of the diet or supplementing the diet with l-Arg, improved weight gain, body composition and grip strength in mice with SCA.

## Discussion

The objective of the present study was to determine the effect of a high-protein diet and increased l-Arg on body composition and grip strength in sickle mice. It was our hypothesis that both a high-protein diet and increased l-Arg would provide additional nutrients that sickle mice might need to improve a characteristically slower rate of weight gain^(^^11^^)^, which would probably result in inadequate LBM and hence less strength for the use of limbs. These results, for the first time, illustrate that dietary supplementation can improve body composition and limb grip strength in transgenic sickle mouse models. The incremental dosage of l-Arg also revealed that increased Arg provided significant improvements in total weight gain and body composition in the TS mouse model. The dosage of Arg that yielded the most significant improvements was the 1·6 % l-Arg diet, which is equivalent to the amount supplied in the high-protein (35 % energy from protein) diet. The TS mice remained anaemic with high reticulocyte counts despite the type of diet consumed (Fig. 3). Therefore it is reasonable to suggest that increased Arg supplied extra energy for improved weight gain and body composition, while continuing to drive erythropoiesis.

Our previous studies showed that S mice need more energy from dietary protein than C mice^(^^11^^)^, concurring with clinical investigations signalling an increased energy need in SCA and corresponding dietary energy shortage^(^^33^^–^^35^^)^. It was also found that the diet with the high proportion of energy derived from protein (35 %) was more beneficial for sickle mice than the standard mouse diet^(^^11^^)^, by improving weight gain and reducing inflammatory biomolecules. The high-protein diet decreased acute-phase and cytokine inflammatory markers after 3 months in S mice^(^^34^^)^, alluding to a possible mechanism to explain decreased infection rates in children with SCA receiving supplements^(^^34^^,^^36^^)^. What remained to be explored were the effects of a high-protein/energy diet on body composition and a better understanding of what component(s) in the high-protein diet may be responsible for improvements. Therefore, the present study was designed as a natural extension of the initial work, to investigate the impact of the high-protein diet and increased l-Arg on these additional nutritional complications that define sickle cell disease. In the present study, S35 mice had higher mean values for total weight gain, LBM and grip strength than S20 mice. The final grip strength for S mice surpassed that for C20 mice. The basis for the small changes noted for grip strength in the C20 mice compared with the other groups cannot be categorically identified, since there are many factors contributing to grip strength. However, since these mice were consuming their optimal diet we did not anticipate any significant improvement in their grip strength. An aspect that has not been explored is physical activity. Throughout the study it was observed that the sickle mice were more active than the control mice. It would be interesting to monitor this behaviour to confirm if the difference in activity contributed appreciably to the difference in grip strength when controlling for diet.

Reports in the literature show that circulating levels of many amino acids are significantly lower than normal for individuals with SCA^(^^12^^,^^37^^,^^38^^)^. Of these, one conditionally essential amino acid of interest is l-Arg, due to its impact on growth^(^^39^^)^ and protein synthesis^(^^40^^)^. We have shown that increased dietary Arg increased plasma l-Arg levels in sickle mice while reducing liver arginase levels, suggesting a shift in Arg metabolism toward less urea production^(^^11^^)^, and possibly more in favour of NO formation, with potential positive effects such as reducing vascular cell/cell adhesion and vaso-occlusion, therefore facilitating increased blood flow, O_2_ distribution and nutrient supply. Other researchers, using a different sickle mouse model (S + S-Antilles) demonstrated that dietary l-Arg supplementation improved physical performance and reasoned similarly that this result could be related to increased NO synthesis, causing more vasodilatation and blood flow by reducing ischaemia in the brain and/or muscle^(^^41^^)^. These findings encouraged the possibility that adding l-Arg to the standard-protein diet could also improve body composition and, hence, grip strength. The results of the present study demonstrate, for the first time, that S and TS mice supplemented with dietary l-Arg improve body composition by dose–response, but not in an expected incremental fashion. The 1·6 % l-Arg diet was associated with the highest mean value for total weight gain and BMD, whereas the 3·2 % l-Arg diet was associated with the largest change in grip strength. Therefore, the results of the dose–response seem to be highlighting differences in l-Arg requirements for diverse physiological processes. Collectively, the present study demonstrates that by supplying additional nutrients required to reduce known protein/energy shortages, key pathological events may be reduced and growth and development improved in SCA.

We have examined the impact of diet on body composition of sickle mice by using the DXA scan method. Children with SCA are reported to have significantly reduced whole-body BMC and significant deficits in LBM^(^^23^^)^. A similar pattern was observed in the present study, in which sickle mice had significantly lower BMD and BMC than the control mice for both standard and enriched diets. Comparing body composition of a separate set of mice at 7d with mice fed the respective diet for 3 months suggested more improvement in the S35 mice compared with S20 mice, suggesting a possibility for catch-up development, if the correct dietary requirement can be determined. To address the question of temporal intra-individual body composition change in transgenic sickle mice, it would be necessary to implement a technique not requiring restraint that would eliminate risk of mortality when the animals are recovering from anaesthesia. Comparison of the TS mouse model with S mice showed that the average BMD, BMC and LBM were higher for TS mice. However, the improvements in body composition for sickle mice on either a high-protein diet or increased l-Arg supplementation support the hypothesis and raise the possibility that nutritional supplements may also improve body composition and clinical status for individuals with SCA.

These results concur with other reports about Arg supplementation, implying a benefit of Arg for improved weight gain and BMD. Arg supplementation has been shown to increase skeletal muscle content, decrease fat^(^^42^^–^^44^^)^, improve weight gain and depress muscle protein turnover^(^^45^^)^ in other animal models. The results of the present study and findings from other reports are encouraging and could be of translational value. The idea that dietary supplementation of macronutrients could provide a widely available health benefit for sickle cell patients warrants further exploration, especially as it is recognised that micronutrients (i.e. vitamins and minerals) alone cannot replace the drain on protein and energy resources associated with the rapid rate of erythrocyte renewal reported in the literature^(^^36^^,^^46^^)^. It will ultimately be important to develop RDA of protein and energy and possibly other nutrients for this group of patients.

In summary, there is often deficiency in several elements of body composition in children and adults with SCA. These results show that feeding a diet with a high proportion of energy derived from protein or adding l-Arg to the normal (control) diet helps improve, but not resolve, nutritional deficiencies of sickle mice. We believe that increased l-Arg or dietary protein beyond that supplied in the standard diet is allowing sickle mice to satisfy some of the increased nutrient demands while facilitating improved growth and repair. The combined results of our previous and current research suggest that the increased-protein diet provides amino acids that are otherwise limited in sickle cell disease for normal growth and body composition. Results from the l-Arg supplementation confirm that increased l-Arg availability and metabolism are part of the mechanism by which the high-protein diet improved body composition in the sickle mice. The use of the two transgenic sickle cell mouse models revealed significantly higher mean values for body composition (i.e. BMD, BMC and percentage body fat) for TS *v.* S mice. However, the pattern of change by diet was similar for both models. Although both the high-protein diet and increased-l-Arg diet improved the physical condition of sickle cell mice, adequate formulation for effective dietary supplementation of SCA patients remains to be studied and reported. These data in sickle mice suggest that a nutritional approach based mainly on increased energy intake and supplementing deficient amino acids could offer significant benefits in the management of sickle cell disease patients and, if proven in the clinical setting, should perhaps become part of the usual treatment regimen.
